# Action-At-A-Distance in DNA Mismatch Repair: Mechanistic Insights and Models for How DNA and Repair Proteins Facilitate Long-Range Communication

**DOI:** 10.3390/biom14111442

**Published:** 2024-11-13

**Authors:** Bryce W. Collingwood, Scott J. Witte, Carol M. Manhart

**Affiliations:** Department of Chemistry, Temple University, Philadelphia, PA 19122, USA; bryce.collingwood@temple.edu (B.W.C.); scott.witte@temple.edu (S.J.W.)

**Keywords:** DNA mismatch repair, DNA repair, MutS, MutL, EXO1, DNA polymerase *δ*

## Abstract

Many DNA metabolic pathways, including DNA repair, require the transmission of signals across long stretches of DNA or between DNA molecules. Solutions to this signaling challenge involve various mechanisms: protein factors can travel between these sites, loop DNA between sites, or form oligomers that bridge the spatial gaps. This review provides an overview of how these paradigms have been used to explain DNA mismatch repair, which involves several steps that require action-at-a-distance. Here, we describe these models in detail and how current data fit into these descriptions. We also outline regulation steps that remain unanswered in how the action is communicated across long distances along a DNA contour in DNA mismatch repair.

## 1. Models for Action-At-A-Distance Along a DNA

In many DNA metabolic processes, such as transcription, replication, recombination, and repair, signaling for DNA processing often occurs across considerable distances along the DNA strand, separate from the actual site where the action is needed. This long-distance communication is crucial for initiating and regulating these processes and typically involves complex and highly ordered protein systems to ensure efficiency.

**Figure 1:**
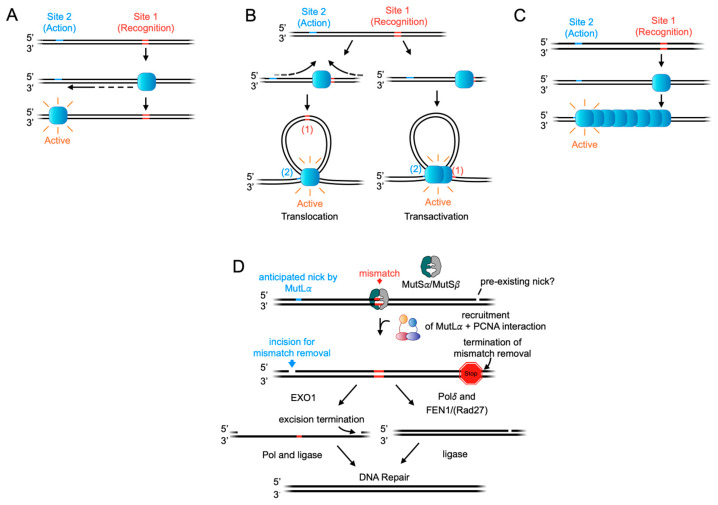
In DNA repair, signals for repair can be widely spaced along the DNA contour, necessitating mechanisms to transmit recognition at one site (site 1) to action at a distant site (site 2). Several models illustrate how this action-at-a-distance can occur. For panels (**A**–**C**), a generic protein is depicted in blue. (**A**) The tracking or sliding model: a protein binds to a recognition site and then moves along the DNA to the site of action. (**B**) DNA looping models: a protein binds to its recognition site and translocates along the DNA, forming an extruded loop. Another variation (transactivation) involves one protein binding to the recognition site and another to the site of action; the dimerization of these two proteins facilitates interactions between the two sites, extruding a loop of DNA. (**C**) Oligomerization model: a protein binds to a recognition site and forms a large oligomeric complex with other copies of itself or additional factors along the DNA, extending to the site of action. (**D**) The model for DNA mismatch repair: action-at-a-distance is essential at several steps to initiate repair and remove mismatches. The repair pathway relies on an incision generated by the MutL*α* protein to excise the mismatch, which can be separated by hundreds of base pairs from the incision site, necessitating communication between the two sites. The mismatch is ultimately removed through excision or DNA synthesis, initiated at the MutL*α* incision site and terminating at a site beyond the mismatch.

To signal across long distances, various generalized strategies have evolved [1]. For example, one mechanism involves a protein recognizing a signal at a distant site (site 1) and moving towards the target site (site 2) along the DNA contour (Figure 1A). Another approach involves DNA being looped, altering distances between sites (Figure 1B). Additionally, the formation of protein oligomers can bridge the gap between two sites (Figure 1C) by forming linear, ordered complexes, constricted complexes that may be ordered or disordered, or by forming a phase that separates condensate or condensate-like structures, which decreases the distance between sites.

While these mechanisms may occur in a variety of DNA metabolic processes, this article reviews how these signaling strategies are used in the context of DNA mismatch repair. We highlight proposed models for how the protein factors involved in DNA mismatch repair use features on the DNA substrate to signal repair events across long distances, ultimately curtailing mutagenesis and maintaining genomic integrity.

## 2. Overview of Mismatch Repair

Mismatch repair is a crucial mechanism that helps maintain genomic stability primarily through correcting errors that occur during DNA replication and limiting mutagenesis (reviewed in [2]). Defects in several mismatch repair proteins are not only linked to increased mutation rates and genomic instability but are also linked to a hereditary cancer predisposition syndrome, Lynch syndrome, in humans [3,4].

The mismatch repair system recognizes and repairs replication errors to prevent mutations from becoming permanent. Much of our understanding of this process comes from its characterization and reconstitution in *Escherichia coli*. In *E. coli*, mismatches are recognized by the MutS homodimer, which binds DNA in an ADP-bound state. Upon detecting a mismatch, MutS exchanges ADP for ATP, adopting a conformation that facilitates the recruitment of the MutL homodimeric complex. MutL acts as a matchmaker, coordinating the activities of MutS and the MutH endonuclease. MutH recognizes hemi-methylated GATC sites, which can be located in several kilobases from the mismatch on either the 5′ or 3′ sides. MutH is activated by interactions with MutL to nick the undermethylated, newly synthesized strand, generally at the closest GATC site, initiating the mismatch repair process. The MutH-generated nick serves as an entry point for exonucleases and/or helicases to remove the mismatch, after which a polymerase fills in the gap, and a ligase seals the strand [5,6].

In vitro studies suggest that MutH-generated nicks on either the 5′ or 3′ side of the mismatch can support repair, though in vivo evidence indicates a preference for 3′ nicks, which are closer to the replication fork [7]. Although the key steps of this process have been understood for decades, certain “action-at-a-distance” steps, such as the communication between a MutS/MutL complex at the mismatch and MutH at distant nicking sites, remain incompletely characterized.

Many components and steps in the *E. coli* mismatch repair pathway are conserved across species. In eukaryotes, there are multiple MutS and MutL homologs that form heterodimeric complexes. In eukaryotic mismatch repair, three MutS homologs (MSH proteins) participate: MSH2, MSH3, and MSH6. MSH3 and MSH6 form separate heterodimers with MSH2, resulting in the MutS*α* (MSH2-MSH6) and MutS*β* (MSH2-MSH3) complexes, which recognize different types of mismatches with overlapping specificities. MutS*α* typically recognizes base–base mismatches and small insertion/deletion loops, while MutS*β* recognizes larger insertion/deletion loops [2,8]. Once a mismatch is recognized, either MutS*α* or MutS*β* recruits a MutL complex.

Like MutS, the MutL family diversifies in eukaryotes, resulting in multiple homologs that form heterodimeric complexes. In mammals, both MutS*α* and MutS*β* recruit the MLH1-PMS2 complex (known as Mlh1-Pms1 in yeast and referred to here as MutL*α*). Unlike *E. coli*, eukaryotes do not possess a MutH homolog or a methyl-directed system for strand discrimination. Instead, MutL*α* acts as a PCNA-stimulated endonuclease, introducing nicks in the newly synthesized DNA strand, often hundreds of base pairs from the mismatch [9,10,11,12,13]. These nicks are essential for mismatch repair [14], which can proceed through multiple pathways (Figure 1D). In one pathway, the MutL*α*-generated nick serves as an entry site for the 5′ to 3′ exonuclease EXO1, which excises a segment of the nascent strand containing the mismatch. DNA polymerase (either *δ* or *ε*) then fills in the gap, and DNA ligase seals the repair [9,15,16]. In an EXO1-independent pathway, the MutL*α*-generated nick initiates strand displacement synthesis by DNA polymerase *δ*, creating a flap structure that is cleaved by a flap endonuclease (FEN1 in humans and Rad27 in yeast) [17]. It should also be noted that even among bacteria, *E. coli* is an outlier, as many bacterial systems are methyl-independent and use an MutL homodimer as an endonuclease.

Despite key differences between methyl-dependent and methyl-independent systems, both processes require long-range communication between the mismatch site and a distant nicking site, with MutL/MutL*α* playing a central role in this coordination.

As discussed above, in *E. coli*, strand discrimination is directed by hemi-methylated DNA, directing MutH to the newly synthesized strand, and mismatch removal predominantly occurs from the 3′ sides. In eukaryotes, the strand discrimination signals are less clear and have been a subject of ongoing research. In vitro mismatch repair reactions using cell extracts or purified proteins demonstrate strand bias only when a pre-existing nick is present on one of the strands [10,11,18,19,20,21,22]. Reconstitution experiments show that if this pre-existing nick is on the 5′ side of the mismatch, repair can proceed without MutL*α*, relying only on MutS*α*, EXO1, and the single-strand binding protein RPA [9,13,23]. If the pre-existing nick is on the 3′ side, repair additionally requires MutL*α*, RFC, and PCNA, suggesting that MutL*α* generates a nick on the 5′ side of the mismatch [24,25,26,27]. In partially reconstituted assays with MutS*α*, MutL*α*, RFC, and PCNA, MutL*α* preferentially nicks the pre-nicked strand, often hundreds of bases from the original nick [10,11]. These observations imply that MutL*α* may distinguish between strands, either alone or in combination with RFC/PCNA, to generate a nick on the 5′ side, enabling mismatch removal from the MutL*α*-generated site.

**Figure 2:**
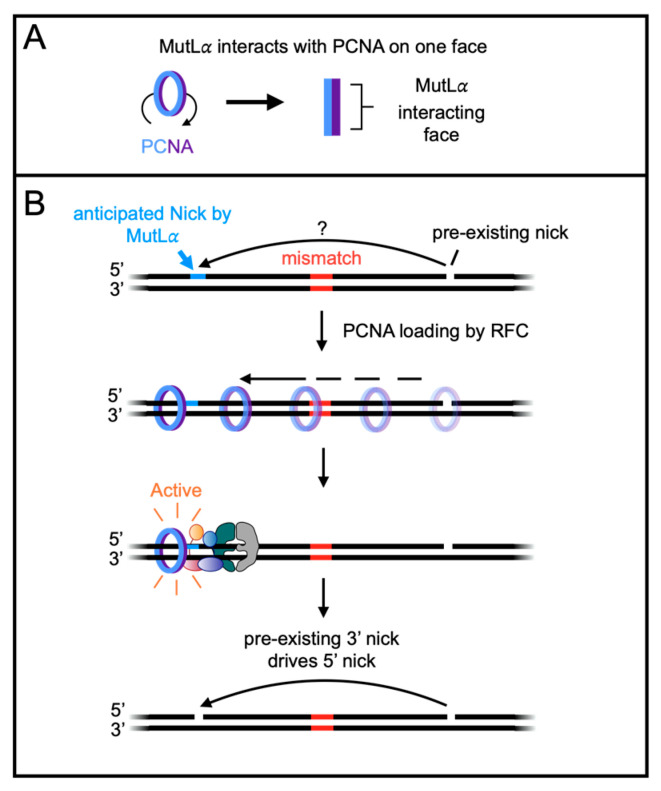
The directionality of mismatch repair systems in vitro and possibly in vivo is guided by pre-existing DNA nicks. (**A**) PCNA has two distinct faces: one that interacts with MutL*α* to stimulate its endonuclease activity and another that does not. (**B**) A model for how a 3′ pre-existing nick could direct an incision on the 5′ side of the mismatch. See the text for details.

The mechanisms underlying MutL*α*’s preference for the pre-nicked strand and bias toward the 5′ side of the mismatch are not fully understood. Studies have shown that PCNA, a clamp protein with two distinct faces, activates MutL*α*’s endonuclease via interactions on one face (Figure 2A) [10,11,28]. RFC can load PCNA at the 3′ end of the pre-existing nick, positioning PCNA so that the face interacting with MutL*α* points toward the pre-existing nick (Figure 2B) (reviewed in [12]). Although PCNA can diffuse along DNA in either direction, evidence suggests that it tracks along a single strand [29]. Through asymmetric interactions with DNA, PCNA may impart strand bias to MutL*α* upon endonuclease activation. However, this model assumes that PCNA moves past the mismatch in the 3′ to 5′ direction. How this occurs is unknown but could potentially be facilitated by interactions between MutS*α* and PCNA.

Although pre-existing nicks in vitro may be artificial in the experimental system, they could also occur in vivo preferentially on newly synthesized DNA. These nicks may arise from Okazaki fragment synthesis, polymerase stalling, or residual nicks from the removal of mis-incorporated ribonucleotides [2,10,11,26,30,31].

**Figure 3:**
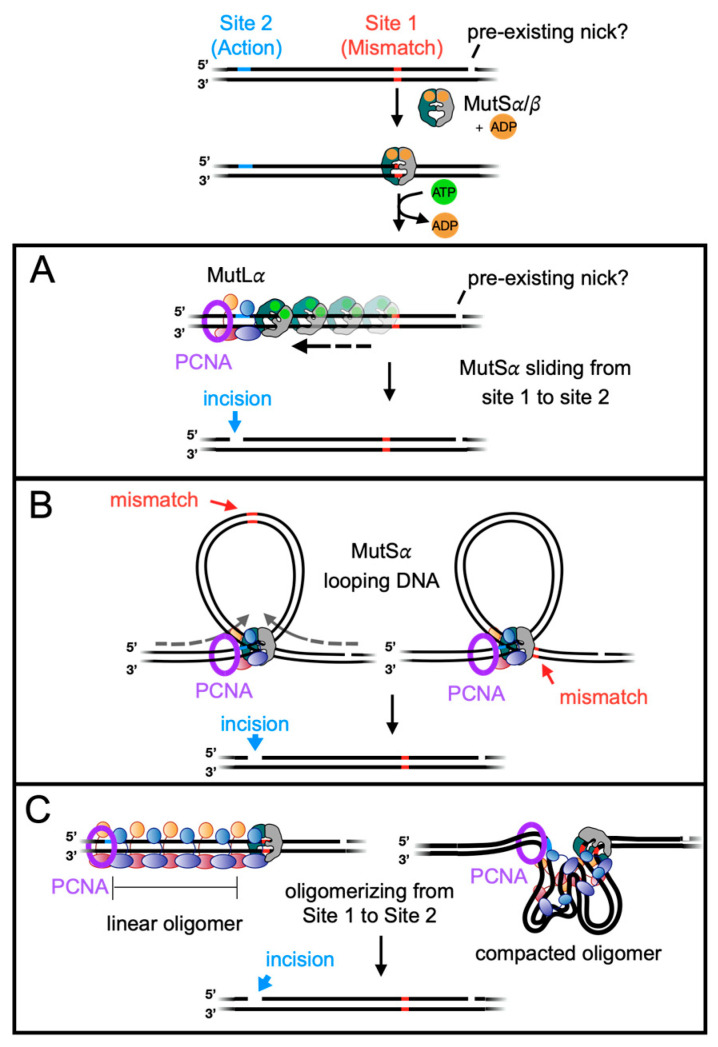
Models for action-at-a-distance between the mismatch and the MutL*α* incision used for mismatch removal. Following the recognition of mismatch by the MutS*α*/*β* complex, MutS*α*/*β* undergoes an exchange of ADP for ATP, enabling an interaction with MutL*α*. (**A**) It has been proposed that upon ATP binding, MutS*α* can function as a sliding clamp, migrating along the DNA to a distant position where it recruits or interacts with MutL*α* to facilitate incision. (**B**) Alternatively, the ATP-bound form of MutS*α* may translocate to extrude a mismatch-containing (heteroduplex) loop, engaging with MutL*α* at a remote site. In another scenario, MutS*α* could remain stationary at the mismatch while forming a complex with MutL*α* bound to the distant site, resulting in the extrusion of a homoduplex loop. (**C**) MutS*α* may also remain associated with the mismatch and interact with an oligomer of MutL*α*, effectively bridging the spatial gap between the mismatch and the MutL*α* incision site. This oligomerization of MutL*α* may induce conformational changes in the DNA structure, facilitating the necessary interactions. Refer to the text for further details.

As outlined, the mismatch repair pathway involves several steps that facilitate long-range communication along the DNA. While some aspects of these signaling processes remain elusive, various models in the literature suggest how these signals are transmitted through protein–protein and protein–DNA interactions. In this review, we explore these models and strategies that facilitate action-at-a-distance signaling steps in DNA mismatch repair. This includes examining how the originating mismatch site acts as a signal for MutL*α* to nick one of the DNA strands at a distant site (Figure 3). Additionally, we discuss models regarding how EXO1 or DNA polymerase *δ* are regulated by their initiation point at the MutL*α* nick and/or other signals on the DNA during mismatch removal.

## 3. Mechanisms for Signaling MutL*α* to Nick DNA Distant to the Mismatch

The first step in eukaryotic mismatch repair is mismatch recognition by MutS*α* or MutS*β*. This recognition triggers the recruitment of MutL*α*, which nicks DNA, which in vitro measurements have suggested can occur at varying distances from the mismatch but can be on the order of several hundred bases away [10,11] (Figure 1D). How mismatch recognition ultimately signals this action-at-a-distance has been the underlying question of many biochemical and biophysical studies, and models invoking all three paradigms described in Figure 1A–C have been proposed.

### 3.1. Sliding Clamp Models to Signal Between the Mismatch and the MutL*α* Nicking Site

Early mechanistic studies investigating how MutS/MutS*α* complexes from bacterial and eukaryotic systems identify mismatches uncovered how these proteins scan DNA in an ADP-bound conformation. Upon mismatch recognition, MutS/MutS*α* exchanges ADP for ATP, which triggers a conformational change. The resultant conformation is capable of acting as a long-lived sliding clamp that can travel along DNA without dissociating in the absence of a free DNA end [32,33,34,35,36,37,38,39]. Many models have been suggested where the MutS/MutS*α* binds to a mismatch, converts into a sliding clamp and then travels to a distant site where it recruits or interacts with MutL/MutL*α*, which can then nick the mispaired DNA strand in eukaryotes, initiating a cascade for mismatch removal and repair (Figure 3A). Variations in this model also include the sliding of the MutL/MutL*α* protein post recruitment by MutS*α* and the MutS*α*/MutL*α* proteins sliding together, away from the mismatch, as a complex [39].

These sliding clamp models involve the MutS complex, typically MutS*α*, recognizing the mismatch, transforming into a sliding clamp, and then moving away from the mismatch. This movement frees the mismatch site from additional cycles of MutS*α* recognition events, creating a signaling gradient where the concentration of MutS*α* and MutL*α* is highest near the mismatch and extends along the DNA toward a distant site. In this model, the mismatch serves as both an initiating signal and an amplifier, establishing a protein concentration gradient that triggers activity at a remote site [32,34,40,41,42]. Despite the predominance of these models in the mismatch repair literature, alternative mechanisms for signaling from the mismatch to a distant nicking event have also been proposed.

Recent studies involving proteins from the thermophilic bacteria *Thermus aquaticus* and the human system suggest that bacterial MutS and mammalian MutS*α* can recognize DNA mismatches, transition to an ATP-bound sliding clamp, and then become arrested on DNA through interactions with either bacterial MutL or MutL*α*, respectively [43,44,45]. In these models, the MutS/MutS*α* complexes not bound to the MutL/MutL*α* complexes can re-recognize the mismatch due to barriers created by arrested complexes and other obstructions on the DNA. In these models, the mismatch still serves as a signal for action, but sliding clamps are not necessarily used to signal over the distance between the mismatch and the MutL/MutL*α* nick site. It should be noted that from the studies with bacterial systems, these models can predict that the nick site must then be generated nearby, as the proteins are confined to a local region [44,45]. However, in the human system, the DNA occluded by proteins extends significantly farther, typically spanning 50–300 base pairs, encompassing both DNA bound by proteins and potentially “missing” DNA due to compaction or looping. This would suggest that the nick may be quite far from the mismatch, which is also predicted by reconstitution experiments and excision tracts [43]. The diversity of models highlights the complexity of signaling in mismatch repair as multiple models coexist to explain how a mismatch can orchestrate repair activity over varying distances along the DNA.

### 3.2. DNA Looping to Signal Between the Mismatch and the MutL*α* Nicking Site

Other models for communication between the mismatch site and the MutL*α* nicking site have involved MutS*α* using ATP to bidirectionally translocate and extrude a loop of DNA, which includes the mismatch (Figure 3B) [46,47]. This model was based on data using the MutH-containing *E. coli* system, suggesting that in an ATP- and mismatch-dependent process, MutS creates a heteroduplex loop, and the presence of the loop is enhanced by MutL which is associated with MutS at the base of the loop [46]. However, the mismatch itself is not suggested to be a part of the protein-bound complex but instead to be a part of the extruded component. Despite this, it still serves as the initiating signal linking the mismatch to a downstream site where MutL is associated with DNA. Although in the *E. coli* system where this was first measured, MutL was not an endonuclease, if such a model is applied to the eukaryotic system, where MutL*α* serves as the endonuclease, the generation of a heteroduplex loop could serve as a signaling channel between the mismatch and the MutL*α* nicking site (Figure 3B).

An additional model has been suggested that invokes DNA looping to signal between the mismatch site and the MutL*α* nick site in eukaryotes. Also, originally suggested for MutS and MutL complexes in *E. coli,* in this model, the MutS complex recognizes the mismatch and binds to the MutL complex at a distant location, effectively linking the two sites through the formation of a DNA loop (Figure 3B) [48]. This process is reminiscent of transactivation mechanisms in transcription, where transcription factors dimerize and create loops in DNA to shorten the distance between enhancer regions and target genes. While this looping model aligns with findings indicating that interactions between the MutL/MutL*α* complexes and MutS/MutS*α* complexes can stabilize MutS/MutS*α* at the mismatch, it contradicts data showing that physical barriers between the mismatch and MutH nicking site disrupt signaling in the *E. coli* system [49]. This suggests that the DNA contour between distant sites must remain intact and plays a crucial role in the signaling mechanism, either directly or indirectly.

### 3.3. MutL*α* Oligomerization Linking the Mismatch and the MutL*α* Nicking Site

To bridge the distance between the mismatch and the site for eukaryotic MutL*α* endonuclease activity, models have also suggested that multiple MutL*α* molecules are recruited during DNA mismatch repair [43,50,51,52,53,54,55]. In these models, MutS*α*/*β* remains stationary at the mismatch [44,45], and multiple MutL*α* copies are recruited to the adjacent DNA duplex. A MutL*α* protein, distant from the mismatch, becomes activated to nick the DNA; thus, the mismatch and the nicking site are physically bridged by the MutL*α* protein (Figure 3C). The formation of these large MutL*α* complexes on DNA is supported by observations that MutL*α* binds to DNA cooperatively [51], preferring larger DNA over smaller substrates in terms of affinity and activity [51,53,55], and is found in excess over MutS*α* in repair complexes [43,52,54]; moreover, in the *E. coli* system, a large tract of DNA becomes resistant to nuclease degradation in a foot-printing study when MutL is added to a reaction with a mismatch and MutS [50].

Interestingly, MutL*α* shares characteristics with proteins that form large nucleoprotein assemblies, including condensates, which often feature intrinsically disordered regions (IDRs) that promote multivalent interactions and phase separation [56]. MutL*α*, a heterodimer composed of MLH1 and PMS2 proteins in mammals, each containing long IDRs, resembles proteins in double-strand break repair, such as 53BP1, PARP1, and XLF-XRCC4. Large assemblies formed by these proteins hold broken DNA ends together for efficient repair [56,57,58]. Similarly, the IDRs of MutL*α* may scaffold protein–protein interactions between MutL*α* copies, facilitating efficient repair.

A large MutL*α* assembly may also explain data investigating trinucleotide repeat expansions where loops and bulges generated by trinucleotide repeat sequences are acted on by mismatch repair erroneously so that the repeat is expanded [59,60,61,62,63,64,65]. In these processes, the eukaryotic MutL homolog complex MutL*γ* (MLH1-MLH3) has primarily been implicated, but MutL*β* (MLH1-PMS1 in mammals, Mlh1-Mlh2 in yeast) and MutL*α* also play roles. Multiple studies into mechanisms promoting expansions have shown that all three MutL complexes may act together as a heterogeneous assembly performing repair in a way that promotes loop expansion [66].

It should be noted that these oligomerization models are not necessarily mutually exclusive to models that invoke DNA looping or sliding clamps. Biophysical experiments have suggested that upon MutL*α* recruitment and the formation of a multimeric complex, the DNA can become compacted or reconfigured [43,67], so a combination of oligomerization and DNA manipulation may serve as a bridging mechanism to connect the mismatch and the site for MutL*α* endonuclease activity (Figure 3C). MutS*α* ATP-associated sliding clamps or ATP-bound MutL*α* sliding clamps may promote the dissociation of the large nucleoprotein assembly after MutL*α* nicks DNA.

## 4. Action-At-A-Distance in Mismatch Removal

After the mismatch is recognized and MutL*α* generates a nick in the newly replicated DNA strand some distance away, the mismatch needs to be removed by either an exonuclease or a polymerase. Both classes are processive and can excise or replicate along significant distances before dissociating. In mismatch repair, since these proteins initiate mismatch removal away from the mismatch, it needs to be established that they travel the appropriate distance along the DNA to ensure mismatch removal.

DNA mismatch repair is unique in that almost all of the actions required to remove and repair a DNA mismatch initiate and terminate at a distance from the mispaired bases, which are removed collaterally, although they signal and restrain the pathway to a region relatively near the mispair. This is significantly different from pathways such as short-patch base excision repair, where the damaged base is removed by a glycosylase, and only a few nucleotides are excised to complete the repair [68]. In contrast to this localized repair mechanism, the mismatch repair process involves more complex signaling across longer distances, underscoring its unique signaling properties.

### 4.1. Signals for Mismatch Removal Are Distant from the Mismatch

**Figure 4:**
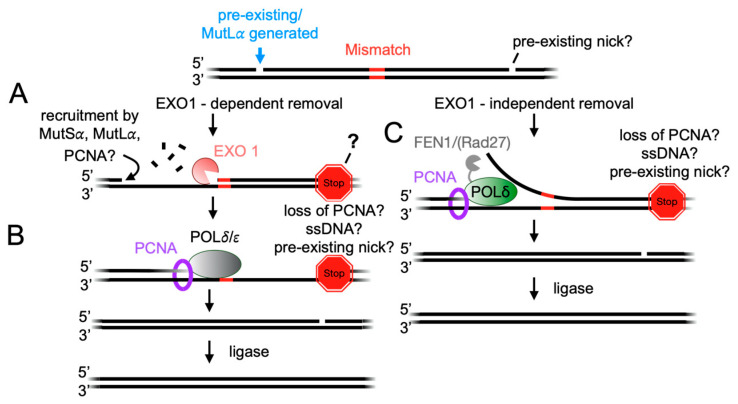
The incision generated by MutL*α* serves as the initiation point for the exonuclease EXO1 or DNA polymerase *δ* to remove the mismatch. The mechanisms by which these proteins are recruited to MutL*α*-generated incisions and regulated to terminate nascent strand removal beyond the mismatch are not fully characterized. (**A**) In the EXO1-dependent mismatch removal pathway, EXO1 is recruited to the incision created by MutL*α*, potentially by repair factors involved in earlier steps, and begins excising DNA in a 5′ to 3′ direction. The activity of EXO1 may be regulated or terminated by factors such as MutL*α* or RPA. (**B**) After the removal of the mismatch-containing nascent strand, DNA polymerase *δ* or *ε* fills in the resulting gap. Gap filling may be regulated beyond the mismatch by collisions with the duplex region, the loss of interaction with PCNA, or the creation of a single-stranded DNA tail through strand displacement, which can reduce polymerase processivity. (**C**) In the EXO1-independent mismatch removal pathway, DNA polymerase *δ*, in conjunction with FEN1 (Rad27), can access the incision made by MutL*α* and begin displacing the error-containing nascent strand. As strand displacement occurs, the polymerase resynthesizes the DNA, correcting the nucleotide mispairing. The termination signal for this process is likely similar to that regulating the gap filling described in panel (**B**).

In both EXO1-dependent and independent mismatch removal pathways, a 5′ nick to the mismatch serves as an initiation point for mismatch removal (Figure 4). This nick may be either pre-existing, potentially created through Okazaki fragment synthesis, polymerase stalling, remaining from the removal of mis-incorporated ribonucleotides, or generated by MutL*α* [2,10,11,26,30,31]. In all of these cases, the nick is some distance away from the mismatch, which is commonly of the order of hundreds of base pairs.

The significance of a 5′ nick relative to directing mismatch removal has been demonstrated through experiments using reconstituted systems with purified proteins or cell-free extracts on plasmid substrates containing either a 5′ or 3′ pre-existing nick [9,10,11,13,18,19,20,21,26,28,69,70,71,72,73]. These studies revealed that 5′ nicks (5′ nick-directed) could be directly used by either EXO1 or DNA polymerase *δ* to remove the mismatch (Figure 4). Conversely, 3′ nicks (3′ nick-directed) serve as signals for MutL*α* to create a 5′ nick via PCNA activation and orientation (Figure 2B). Mismatch removal then proceeds through the 5′ nick, using either EXO1 or DNA polymerase *δ* (Figure 1D) [25]. In both 5′ and 3′ nick-directed systems, the repair rate declines as the distance between the pre-existing nick and mismatch increases, though repair still occurs even when the distance is around 1000 base pairs, indicating that the signal for mismatch removal can be transmitted over considerable distances [70].

### 4.2. Initiating Excision at a MutL*α* Nick and Terminating Beyond the Mismatch

In the EXO1-dependent pathway, EXO1 generates a single-stranded DNA gap that extends from a 5′ pre-existing nick or a MutL*α* nick site to a point beyond the mismatch. The length of these tracts varies by organism; for instance, tracts in *S. cerevisiae* are significantly longer than those in human systems, although both are at least ~200 nucleotides in length [9,13,23]. These findings suggest that the MutL*α* nick serves as an initiating signal for excision, while a site on the flanking side of the mismatch acts as a termination signal, indicating that these sites can be separated by hundreds of base pairs (Figure 1D, Figure 4A).

Beyond the nick itself, other factors such as MutS*α* and MutL*α* may also signal the recruitment and initiation of excision by EXO1. The MSH2 subunit of MutS*α*/*β* and the MLH1 subunit of MutL*α* are reported to contain EXO1 binding motifs, which are believed to recruit the exonuclease to the nick site either independently or collaboratively [74,75,76,77,78,79,80,81]. However, some data suggest that MutL*α* may actually limit excision by EXO1 [13,23,76,82,83]. This raises the possibility that MutL*α* plays a dual role in EXO1 regulation, acting as both a recruitment signal at the 5′-end of a nick and a termination signal to restrict excision beyond the mismatch.

Recent investigations into the interaction between MutS*α* and EXO1 via a conserved motif in EXO1 suggest redundancy in EXO1’s interactions with both MutS*α* and MutL*α* [76]. To eliminate EXO1-dependent mismatch repair, it was found that both the MutS*α* and MutL*α* interaction sites with EXO1 needed to be abrogated simultaneously. This indicates that either MutS*α* or MutL*α*, or both proteins working together, may recruit EXO1 to nicks located 5′ from the mismatch. Furthermore, it is plausible that MutS*α* initiates the excision process while MutL*α* terminates it through physical interactions. This model aligns with evidence showing that MutS*α* enhances the length of EXO1 excision tracts [9] while human MutL*α* appears to limit EXO1 excision [13,23,83].

Reconstitution experiments and studies using extracts have also demonstrated that the single-stranded binding protein replication protein A (RPA) plays a crucial role in mismatch repair. RPA binds to the single-stranded DNA produced by EXO1 excision, protecting it from further damage [13,23,24,84]. Interestingly, in in vitro studies, RPA exhibits both stimulatory and inhibitory effects on EXO1 activity. At low concentrations, RPA binds to nicked heteroduplex DNA and enhances EXO1’s activity on the substrate, suggesting that it may signal EXO1 to initiate excision at the nick. Conversely, at high concentrations, RPA appears to limit EXO1 excision. This indicates that when a long stretch of single-stranded DNA generated behind EXO1 becomes coated with RPA, it may serve as a termination signal for EXO1 to cease excision once sufficient single-stranded DNA has been generated [13,23,24].

Further mechanistic research is essential to clarify the regulatory mechanisms that govern the initiation and termination of EXO1 excision, as well as the roles of other mismatch repair factors in this process. Although EXO1 deletions in model systems do not exhibit dramatic mutator phenotypes [15,22,85], there is evidence that other mismatch removal processes are largely compensatory and primarily active in the absence of EXO1 [52]. This underscores the need for a clearer understanding of how EXO1 is regulated by other mismatch repair factors during mismatch removal. Additionally, recent work identifying a conserved motif for MSH2 interactions—along with its presence in EXO1 and other factors involved in EXO1-dependent mismatch repair—opens avenues for further mechanistic exploration [76].

### 4.3. Initiating and Terminating DNA Resynthesis

To complete DNA repair in the EXO1-dependent pathway, the single-stranded gap must be filled in by a DNA polymerase. Reconstitution and genetic assays have shown that both the leading and the lagging strand polymerases can fill in this gap. These proteins are suggested to initiate gap filling at the 3′ OH at one side of the gap and to terminate synthesis at the other end (Figure 4B) [13,23,24,84,86]. Experiments using mismatched plasmids to probe the EXO1-dependent pathway, incubated with nuclear extracts with and without the PCNA inhibitor protein, p21, have shown that PCNA is needed both to initiate mismatch repair by way of activating MutL*α* and to promote the resynthesis of DNA [87]. In DNA replication, PCNA is used as a processivity factor, enhancing the processivity and strand displacement activities of DNA polymerases. This function, along with the observation that PCNA is required for DNA resynthesis in mismatch repair, suggests that PCNA is likely playing a similar processivity-enhancing function for polymerases filling in the EXO1-generated gap during the mismatch removal step. PCNA plays a role in the initial steps of mismatch repair, potentially acting as a strand discrimination signal for both MutS*α* and MutL*α*. As a result, PCNA is already present on the mismatched DNA substrate before excision begins. Since physical interactions between MutL*α* and PCNA are necessary to activate MutL*α*’s endonuclease activity [88], PCNA is likely to be located near the MutL*α*-induced nick that initiates DNA resynthesis. This positioning may serve as an additional initiation signal for DNA polymerase *δ* or *ε*. Although both DNA polymerase *δ* and *ε* have been shown to be able to resynthesize DNA that has been excised during DNA mismatch repair, DNA polymerase *δ* is thought to primarily be responsible for this process. Because DNA polymerase *δ* has strand displacement activity, this resynthesis step must be regulated past the mismatch so that activity is limited. This regulation has largely been unexplored in the context of mismatch repair, but in studies investigating DNA polymerase *δ*’s activities in lagging strand replication where it serves as the primary polymerase, it has been suggested that the presence of long single-stranded 5′ flaps can limit strand displacement synthesis activity [89]. This suggests that in DNA mismatch repair, once DNA polymerase *δ* has filled in the gap created by mismatch excision and it encounters duplex DNA, it might displace strands for some distance but be inhibited by the creation of a flap. Structural and biochemical work investigating *E. coli* MutL has suggested that the protein forms a stable interaction with 3′-resected ends, and this activity can inhibit DNA polymerases from using 3′-resected ends as the primer termini [86]. Although this polarity is not congruent with terminating gap filling and colliding with another duplex region, a more general DNA end-binding interaction could also limit strand displacement activity. Another hypothesis is that PCNA has been shown to stimulate strand displacement activity [89]. It is possible that PCNA becomes displaced from the gap-filling polymerase near the end of the gap by an unknown mechanism, and the loss of the processivity factor aids in terminating replication. Additionally, the DNA polymerase *δ* that is involved in mismatch repair might encounter other barriers, such as newly deposited nucleosomes or the replication fork, which might limit or terminate the polymerase.

### 4.4. Initiating and Terminating Mismatch Removal with DNA Polymerases

Studies where EXO1 was deleted using baker’s yeast as a model have suggested that there are compensatory pathways for mismatch removal, and the repair process is not completely reliant on EXO1. This observation has led to the discovery of EXO1-independent mismatch removal pathways, which include the removal of the mismatch from the MutL*α*-generated nick using the stand-displacement synthesis activity of DNA polymerase *δ* in combination with a flap endonuclease—Rad27—in yeast and FEN1 in humans [16,17,90,91] (Figure 1D). Similarly to EXO1-dependent mismatch repair, this pathway requires that a DNA polymerase *δ*-Rad27 (FEN1) complex be recruited to the 5′ nick as an initiation point and to terminate replication at a site beyond the mismatch (Figure 4C). Similarly to the resynthesis of excised mismatched DNA, how DNA polymerase *δ* is initiated and terminated in this pathway has largely been unexplored, but it is likely recruited and terminated by similar mechanisms regardless of whether the protein is performing gap-filling or strand displacement mismatch removal.

Another EXO1-independent mismatch removal pathway has been suggested where a DNA strand break on the 3′ side of the mismatch can be used as an initiation site for mismatch removal using the 3′ to 5′ exonuclease proofreading activity of DNA polymerase *δ* or *ε* [2]. This single-strand break or pre-existing nick could be present due to Okazaki fragment synthesis on the lagging strand, discontinuous DNA replication on the leading strand, or due to the removal of mis-incorporated ribonucleotides. How exactly these breaks serve as initiation signals explicitly for DNA polymerase exonuclease proofreading functions is unknown. It has been shown that a replicating polymerase can excise a mis-incorporated nucleotide up to seven bases behind the primer terminus, highlighting that mismatch removal by a proofreading polymerase could be possible [92]. Despite this removal pathway being proposed, it has never been observed in vitro, and repair using a pre-existing nick on the 3′ side of the mismatch has largely been suggested to proceed through this nick being used as a strand discrimination signal for MutL*α* to generate an additional 5′ nick to the mismatch.

## 5. Conclusions

DNA mismatch repair employs various models of action-at-a-distance, given that the initiation and termination of mismatch removal occur at distances from the mismatch itself. The dysregulation or failure of DNA mismatch repair is linked to increased mutagenesis and hereditary cancer syndromes in humans, emphasizing the critical need to understand how these action-at-a-distance signals are both initiated and regulated. The separation of initiating signals, such as the mismatch, from the sites of mismatch removal—often hundreds of base pairs apart—complicates our understanding of the underlying signaling mechanisms, which likely involve complex interactions among multiple repair factors. Reconstructing these mechanistic signals is challenging, necessitating a multifaceted approach that incorporates genetic, molecular, structural, biophysical, and biochemical data.

The complex nature of these signaling processes leaves many questions unanswered regarding the regulation within DNA mismatch repair, as outlined in this review. Furthermore, the features that characterize mismatch repair make it an excellent model system for exploring the broader principles of action-at-a-distance signaling utilized by proteins. Insights gained from studying mismatch repair could inform our understanding of other critical DNA metabolic pathways, including transcription, replication, and various DNA repair mechanisms.

## Data Availability

No new data were created or analyzed in this study. Data sharing is not applicable to this article.
